# Characterizing animal anatomy and internal composition for electromagnetic modelling in radar entomology

**DOI:** 10.1002/rse2.94

**Published:** 2018-08-29

**Authors:** Djordje Mirkovic, Phillip M. Stepanian, Charlotte E. Wainwright, Don R. Reynolds, Myles H. M. Menz

**Affiliations:** ^1^ Cooperative Institute for Mesoscale Meteorological Studies University of Oklahoma Norman Oklahoma 73072 USA; ^2^ Computational and Analytical Sciences Department Rothamsted Research Harpenden Hertfordshire AL5 2JQ United Kingdom; ^3^ Corix Plains Institute University of Oklahoma Norman Oklahoma 73019 USA; ^4^ Natural Resources Institute University of Greenwich Chatham Kent ME4 4TB United Kingdom; ^5^ Institute of Ecology and Evolution University of Bern 3012 Bern Switzerland; ^6^ School of Biological Sciences The University of Western Australia Crawley 6009 Western Australia Australia

**Keywords:** Electromagnetic modelling, entomological radar, Insect RCS, insects, migration, radar cross‐section, weather surveillance radar

## Abstract

The use of radar as an observational tool in entomological studies has a long history, and ongoing advances in operational radar networks and radio‐frequency technology hold promise for advances in applications such as aerial insect detection, identification and quantification. Realizing this potential requires increasingly sophisticated characterizations of radio‐scattering signatures for a broad set of insect taxa, including variability in probing radar wavelength, polarization and aspect angle. Although this task has traditionally been approached through laboratory measurement of radar cross‐sections, the effort required to create a comprehensive specimen‐based library of scattering signatures would be prohibitive. As an alternative, we investigate the performance of electromagnetic modelling for creating such a database, focusing particularly on the influence of geometric and dielectric model properties on the accuracy of synthesized scattering signatures. We use a published database which includes geometric size measurements and laboratory‐measured radar cross‐sections for 194 insect specimens. The insect anatomy and body composition were emulated using six different models, and radar cross‐sections of each model were obtained through electromagnetic modelling and compared with the original laboratory measurements. Of the models tested, the prolate ellipsoid with an internal dielectric of homogenized chitin and hemolymph mixture best replicates the measurements, providing an appropriate technique for further modelling efforts.

## Graphical abstract

The manuscript outlines calculations aimed for creating comprehensive insect specimen library of radar cross sections (RCS). Using the computational electromagnetic (CEM) approach, we compare six different model topologies and two compositions for comparison with the published measurement results for almost 200 insect specimens.

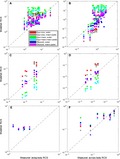

## Introduction

It has been over 60 years since Crawford (1949) first identified the presence of insects during radar observations and, in a remarkably prescient comment, he suggested that special‐purpose radars might thereby provide the capability of remote insect monitoring. Realizing the benefits of this new observational technique for characterizing aerial fauna aloft occurred sometime later – the first *dedicated* radar‐entomological field studies took place in 1968 (Schaefer 1976). Since the time of these pioneering studies, radars of several configurations (e.g. scanning, profiling, transecting and tracking), wavelengths and transmitter powers have been used in hundreds of entomological research studies (over 250 publications containing significant radar entomology content are listed on the Radar Entomology Website [http://radarentomology.com.au/bibliography/]). Many of these studies have used specially developed ‘entomological’ radars (Drake and Reynolds 2012), which have acquired quantitative estimates of insect activity unobtainable by any other means, and revealed unanticipated behavioral phenomena (e.g., Hu et al. 2016; Reynolds et al. 2016, 2017; Wainwright et al. 2017). In parallel efforts in the atmospheric sciences, airborne insects were also detected by early meteorological research radars and, after initial controversy over their biological origin (see Chapt. 15 in Drake and Reynolds 2012), atmospheric research radars have significantly contributed to our knowledge of insect migration (e.g. Russell and Wilson 1997; Geerts et al. 2006; Browning et al. 2011; see Chapt. 11 and 15 in Drake and Reynolds 2012).

Since the 1990s, special‐purpose entomological radars have principally used a ‘ZLC configuration’ (zenith‐pointing linear‐polarized (narrow‐angle) conical scan) (Chapt. 5 in Drake and Reynolds 2012; Drake 2014, 2016), which lend themselves to autonomous operation. These radars interrogate *individual* insects (above ~ 2 mg mass) as they transit the vertical beam, and provide (inter alia) information on the size and the shape of the target.

Despite the specialized utility of these units, their relatively small surveillance area restricts large‐scale monitoring, and the ongoing costs associated with deployment, maintenance and data analysis from networks of these radars may be outside of current practical capability. Consequently, there has been much recent emphasis on using operational networks of weather surveillance radars (WSRs) for regional and continent‐wide monitoring of flying animals (Gauthreaux et al. 2008; Chilson et al. 2011; Dokter et al. 2011; Kelly et al. 2012; Shamoun‐Baranes et al. 2014; Bauer et al. 2017). The great advantage here is that extensive radar networks are already in existence and that their operation and maintenance costs do not have to be borne by the biological research community. Additionally, WSR networks are currently being upgraded to dual‐polarization systems, and this technology will greatly improve our ability to distinguish birds, bats and insects, and different forms of precipitation in routine weather radar observations (Chandrasekar et al. 2013; Melnikov et al. 2014, 2015; Stepanian et al. 2016). Initial studies have revealed vast potential for entomological application, including taxonomic identification, behavioral characterization, surveillance and monitoring of broad‐scale movement (Rennie et al. 2010; Leskinen et al. 2011; Melnikov et al. 2015; Boulanger et al. 2017; Westbrook and Eyster 2017), but significant foundational work is still required before widespread adoption of these capabilities is possible.

The capacity of a radar target, such as a flying animal, to reflect signals back in the direction of the radar receiver is determined by its backscattering radar cross‐section (RCS) (Knott 2012). This property will depend on the target's size and shape, the dielectric material within the target, the radar wavelength, aspect angle, polarization (orientation of the E‐field) and any time‐dependent motions of parts of the body with respect to each other (due to, for example, wing beating) (Drake and Reynolds 2012). Radar cross‐sections of animals are difficult to measure (see, for example Mirkovic et al. 2016 for some of the issues), and while laboratory or field measurements of insect RCSs have been made (e.g. Riley 1985; Wolf et al. 1993; Hobbs and Aldhous 2006; Drake et al. 2017), insect aerial fauna are very diverse and no direct RCS measurements exist for most species. Moreover, most of the existing measurements pertain to a particular viewing angle (e.g. a ventral view) or wavelength (usually X‐band), while biological data obtained from WSRs would involve a range of aspects, and other wavelengths (C‐ and S‐bands). Finally, we note that routinely available WSR data on high‐altitude animal movements will not necessarily be accompanied by ancillary (e.g. visual) observations or associated trapping studies, which often form part of research campaigns with special‐purpose ornithological or entomological radars. It is therefore important to be able extract the maximum amount of target identification information from the weather radar data itself.

Existing polarimetric Doppler weather radar networks have the potential to be used as quantitative surveillance networks for high‐flying insects, but for this capability to be realized will require full RCS signatures for the numerous insect taxa likely to be present at altitude. Moreover, these RCSs will be needed for the range of different orientations wavelengths and polarizations that make up operational radar networks. The only practical way to gain RCSs across such a range of insect species, radar wavelengths and polarizations in a standardized way is via simulation. The above‐mentioned ‘ZLC configuration’ entomological radars provide an estimate of the RCS size and two shape parameters for each successfully analysed target. Clearly, any convenient means of generating RCS size and shape characteristics of known insect species, which can be *directly* compared with the outputs from these radars, would be invaluable.

Our goal is to determine the performance and limitations of electromagnetic modelling techniques for emulating the scattering characteristics of insects, thus determining the most acceptable modelling method for creating a comprehensive database of insect scattering signatures. To achieve this aim we test six different anatomy and body composition models of insect specimens using body measurements and laboratory‐measured RCSs from a published database. The RCSs for each model are calculated using electromagnetic modelling software and compared against the laboratory‐measured values for each insect specimen.

## Materials and Methods

### Overview

The modelling software used herein is the WIPL‐D implementation of the method of moments (described by Mirkovic et al. 2016). Within this framework, a three‐dimensional object is represented by a set of interconnected plates which define the shape of the object. The internal composition of the object is defined in terms of dielectric permittivity (Mirkovic et al. 2016). An incident electromagnetic field is also defined by the user, which allows for investigation of scattering that results from different radar wavelengths, polarizations and at different incident aspect angles. The currents induced across each plate, due to the incident wave, are calculated from boundary conditions, and inserted into Maxwell's equations, yielding the resultant scattered electromagnetic field. This process can be repeated to produce a comprehensive set of scattering characteristics across a range of incident aspect angles, wavelengths and polarizations. Moreover, this software‐based technique is reproducible, enabling replication of these methods on other specimens with confident inter‐comparability.

There are two major considerations when creating the digital model using this technique. The first is the level of anatomical detail that is included in the digital representation of the animal. In an extreme case, all external physical characteristics could be replicated (e.g., as in Mirkovic et al. 2016), but this is a tedious process and may not be necessary for small insect targets. The other extreme is to omit all anatomy, reducing an animal to a sphere of some fixed radius. A popular compromise is the use of a prolate spheroid to emulate the cigar‐like body shape of flying insects (e.g., Melnikov et al. 2015). The second consideration is the dielectric composition of the digital model. For example, the model can be defined as being water, muscle, insect chitin or some other dielectric medium (Chapt. 4 in Drake and Reynolds 2012). We surmise that some combination of a general body geometry and dielectric composition should yield scattered waves that are representative of true insect characteristics.

### Reference dataset

To determine the best model configuration for emulating the scattering characteristics of insects, we use a collated dataset of ventral X‐band measurements from 194 insect specimens at two orthogonal polarization alignments as a reference against which to compare modelling results (Drake et al. 2017). These 194 specimens span a diverse set of species, masses, and body shapes, ranging from a 1.8‐mg diamondback moth (*Plutella xylostella*) to a 4.12‐g migratory locust (*Locusta migratoria*). From these data, we consider each specimen's physical measurements of body length (along the anteroposterior axis), body width (along the lateral axis), and body mass. Note that measurements along the dorsoventral axis were not collected for these specimens. We also use the measured RCS of each specimen, taken from the ventral viewing angle (i.e., below, looking upwards) with the polarization of the incident electric field parallel to the insect anteroposterior axis (along‐body RCS, herein), as well as parallel to the insect lateral axis (across‐body RCS). To facilitate comparisons, we group the 194 specimens into eleven taxonomic categories: grasshoppers and locusts (Orthoptera; Acrididae); green lacewings (Neuroptera: Chrysopidae); nymphalid butterflies; noctuid moths; pyralid and plutellid moths; geometrid moths; craneflies (Diptera; Tipulidae); hoverflies (Diptera: Syrphidae); curculionid and carabid beetles (Coleoptera); ladybird beetle (Coleoptera: Coccinellidae); honeybees and wasps (Hymenoptera) (see Table 1).

**Table 1:** The minimum, mean and maximum RCS percent errors for each of the six model categories (see Methods) and eleven taxon groups. RCS, radar cross‐section

Group (number of specimens)		Model 1 (Equi‐size, water‐filled) RCS % error (min, mean, max)	Model 2 (Equi‐size, insect‐filled) RCS % error (min, mean, max)	Model 3 (Equi‐mass, water‐filled) RCS % error (min, mean, max)	Model 4 (Equi‐mass, insect‐filled) RCS % error (min, mean, max)	Model 5 (Ellipsoid, water‐filled) RCS % error (min, mean, max)	Model 6 (Ellipsoid, insect‐filled) RCS % error (min, mean, max)
Orthoptera (47)	Along body	−43.5%, 183.3%, 1138.5%	−56.8%, 133.9%, 801.3%	−41.6%, 459.3%, 2318.7%	−25.8%, 263.5%, 1642.4%	−65.6%, 58.7%, 636.6%	−64.1%, 63.0%, 647.8%
	Across body	−97.4%, 187.2%, 3029.6%	−97.2%, 170.5%, 3123.0%	−89.8%, 1102.8%, 9419.3%	−94.0%, 602.1%, 5756.7%	−96.6%, 25.4%, 1290.5%	−96.1%, 102.1%, 2280.3%
Neuroptera – lacewings (6)	Along body	274.6%, 575.6%, 1037.2%	115.9%, 296.3%, 650.3%	392.0%, 589.9%, 741.0%	64.2%, 132.7%, 233.4%	322.9%, 579.0%, 905.8%	84.1%, 191.7%, 388.7%
	Across body	−63.9%, −29.8%, 80.9%	−63.7%, −28.4%, 84.8%	−59.6%, −26.5%, 41.4%	−80.4%, −59.5%, −9.0%	−60.1%, −31.1%, 63.2%	−67.6%, −43.7%, 4.3%
Lepidoptera – nymphalid butterflies (10)	Along body	−21.5%, 556.0%, 3926.8%	2.8%, 628.4%, 4408.1%	−23.2%, 670.7%, 4826.4%	−1.0%, 464.8%, 2755.4%	−29.6%, 739.6%, 5481.6%	−8.9%, 701.8%, 4532.4%
	Across body	−35.1%, 572.9%, 3667.6%	−68.2%, 424.5%, 2708.6%	−39.4%, 141.7%, 522.5%	−83.6%, −14.5%, 96.6%	−25.3%, 206.9%, 847.1%	−74.4%, 81.5%, 751.0%
Lepidoptera – noctuid moths (81)	Along body	−9.5%, 302.9%, 3915.6%	−32.3%, 244.2%, 3256.3%	−19.0%, 198.4%, 2103.5%	−52.6%, 86.8%, 748.0%	−8.1%, 328.4%, 4290.1%	−11.3%, 260.2%, 3427.0%
	Across body	−61.5%, 739.9%, 12298.6%	−80.7%, 718.9%, 10902.1%	−68.9%, 100.4%, 791.9%	−91.8%, −13.2%, 232.1%	−61.7%, 323.9%, 7389.0%	−82.0%, 173.3%, 5193.1%
Lepidoptera – pyralid/plutellid moths (6)	Along body	73.7%, 326.0%, 719.4%	11.5%, 201.2%, 449.6%	−30.9%, 129.9%, 313.3%	−73.6%, −1.0%, 80.5%	30.0%, 223.5%, 505.0%	−45.5%, 65.7%, 193.9%
	Across body	−89.4%, −41.4%, 51.0%	−89.2%, −40.6%, 54.0%	−94.0%, −63.1%, 2.4%	−96.3%, −76.5%, −34.4%	−88.6%, −52.1%, 28.2%	−90.5%, −61.0%, 4.8%
Lepidoptera – geometrid moths (5)	Along body	693.0%, 2567.6%, 5551.7%	407.6%, 1692.4%, 3729.1%	68.6%, 319.6%, 585.6%	−27.2%, 73.1%, 185.5%	433.9%, 1121.8%, 2409.4%	124.2%, 402.6%, 926.4%
	Across body	−47.2%, 112.3%, 463.6%	−65.4%, 68.8%, 251.3%	−85.6%, −66.2%, −54.9%	−90.8%, −77.2%, −69.0%	−74.5%, 0.5%, 92.5%	−76.8%, −16.1%, 57.3%
Diptera – craneflies (4)	Along body	369.0%, 687.8%, 1271.5%	98.0%, 552.2%, 1366.4%	678.6%, 811.3%, 1016.4%	245.7%, 331.4%, 413.1%	591.2%, 823.5%, 1261.5%	204.0%, 376.7%, 509.1%
	Across body	−92.3%, 233.9%, 696.2%	−91.9%, 133.7%, 362.6%	−79.6%, 115.1%, 408.2%	−85.9%, 43.0%, 250.9%	−88.2%, 112.0%, 190.3%	−89.5%, 83.7%, 149.4%
Diptera – hoverflies (10)	Along body	763.1%, 1730.8%, 3713.3%	425.7%, 1063.5%, 2487.4%	−54.9%, 46.5%, 263.2%	−78.6%, −35.4%, 39.3%	284.6%, 618.8%, 1300.5%	45.9%, 200.3%, 424.4%
	Across body	0.8%, 398.2%, 758.5%	8.2%, 145.2%, 309.5%	−92.1%, −68.0%, −48.4%	−94.6%, −79.5%, −69.3%	−41.3%, 24.3%, 82.1%	−53.3%, 3.6%, 50.0%
Coleoptera – curculionid & carabid beetles (10)	Along body	−63.3%, 298.6%, 1482.0%	−67.0%, 254.9%, 1222.3%	−59.2%, 16.5%, 295.2%	−77.6%, −42.8%, 109.5%	−62.0%, 171.8%, 916.5%	−71.7%, 74.4%, 525.1%
	Across body	−81.7%, 200.4%, 877.4%	−80.5%, 198.8%, 903.3%	−79.9%, −14.3%, 190.9%	−86.3%, −50.6%, 97.6%	−81.0%, 77.9%, 552.8%	−83.3%, 39.6%, 450.5%
Coleoptera – ladybird beetles (8)	Along body	280.1%, 510.1%, 721.7%	151.6%, 360.8%, 666.6%	−75.2%, −56.2%, −9.0%	−90.2%, −82.4%, −72.4%	−10.4%, 54.8%, 135.0%	−33.6%, 13.5%, 74.0%
	Across body	378.2%, 510.4%, 870.2%	118.9%, 310.2%, 609.9%	−82.0%, −62.3%, −46.2%	−88.7%, −83.2%, −74.3%	−5.2%, 25.4%, 84.9%	−25.1%, 1.5%, 47.6%
Hymenoptera – honeybees & wasps (7)	Along body	21.8%, 159.9%, 542.4%	32.0%, 137.2%, 409.3%	22.6%, 196.1%, 780.6%	32.5%, 167.9%, 687.9%	20.2%, 153.6%, 506.7%	35.6%, 155.3%, 534.5%
	Across body	−19.4%, 79.0%, 296.1%	−26.8%, 50.1%, 183.4%	−22.1%, 169.6%, 846.4%	−47.5%, 52.6%, 440.7%	−15.3%, 110.0%, 480.8%	−43.9%, 49.3%, 364.1%
All taxa together (194)	Along body	−63.3%, 440.5%, 5551.7%	−67.0%, 326.0%, 4408.1%	−72.2%, 283.0%, 4826.4%	−90.2%, 133.7%, 2755.4%	−65.6%, 308.7%, 5481.6%	−71.7%, 206.2%, 4532.4%
	Across body	−97.4%, 445.4%, 12289.6%	−97.2%, 399.2%, 10902.1%	−93.94%, 309.5%, 9419.3%	−96.3%, 119.3%, 5756.7%	−96.6%, 162.1%, 7389.0%	−96.1%, 103.1%, 5193.1%

### Model variations

To determine the most appropriate model configurations, we test three body geometries and two internal compositions. In increasing order of complexity, the three body geometries are defined as the following. The first uses the measured body length and width of each insect specimen to define major and minor axis sizes of a prolate spheroid (equi‐size prolate spheroid). The performance of the equi‐size prolate spheroid model is investigated in terms of percentage mass error as described below. The second uses the measured body length and width of each specimen to define the axis ratio of a prolate spheroid, but the spheroid is scaled in absolute size until its mass, based on the density of the internal composition, equals that of the measured specimen. This model is referred to as the equi‐mass prolate spheroid and its performance is assessed via percentage size error below. The third uses the measured size of the insect specimen's body length and width, combined with the measured mass, to define three independent axes of a prolate ellipsoid. From the measured length (*l*), width (*w*), mass (*m*) and an assumed internal density (*d*), the dorsoventral height of the prolate ellipsoid is defined as,

(1) h=(6m)/(πdlw)

We note here that *l*,* w* and *h* are the measured diameters of each specimen along the three axes, and not the corresponding radii. By providing this third degree of freedom, these ellipsoids are equal in mass, length and width to the measured insect specimens.

For each of the three geometries described above we consider two possible internal compositions, to give six total models. The first, and simplest, is water, having an X‐band dielectric permittivity of 60.3 − *j*33.1 and density of 1 g/cc. The second substance is a homogenized blend of lesser grain borer beetles (*Rhyzopertha dominica*), described by (Nelson et al. 1998), and has permittivity of 34.3 − *j*18.6 and density of 1.26 g/cc. The primary divergence of this substance from water is due to the chitin content of insect exoskeletons, which is less reflective at radio frequencies and denser than pure water. For brevity, we will refer to this homogenized substance as “insect paste”.

### Comparison methods

Before conducting any electromagnetic scattering analysis, it is useful to consider how well each model recreates the physical specimen that it is meant to emulate. That is, when creating an equi‐size model based on physical dimensions, how accurate is the resulting mass of the model compared to the physical specimen? Similarly, when constructing equi‐mass models, how do the resulting sizes compare to specimen measurements? Taking the measured values of insect mass, length and width as the referenced truth, we define the model percent error of a given specimen attribute as

(2) Percent error=100∗(modeled‐measured)/measured.

Use of percent error, as opposed to absolute error, is intended to provide normalization, such that the model performance can be compared among diverse size ranges. The along‐ and across‐body RCS values calculated using the electromagnetic modelling software are also compared to the corresponding laboratory‐measured values measurements using (2).

## Results

### Comparison of physical attributes

**Figure 1:**
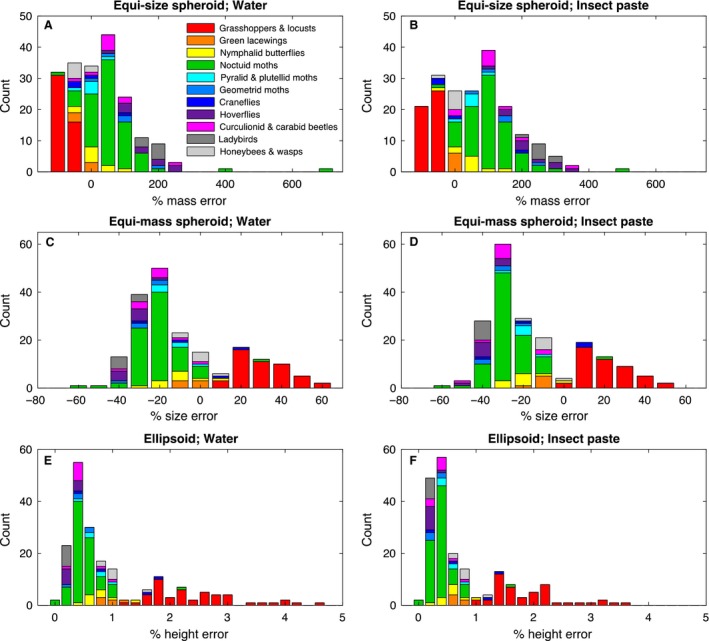
Percentage errors arising from comparisons between modeled geometries and measurements of physical specimens from various insect taxa (see text).

When taking the specimen length and width as the fixed metrics for making an equi‐size prolate spheroid model, the resulting model mass will often deviate from the measured specimen mass (Fig. 1A and B). In this case, positive percent errors indicate equi‐size models that result in mass overestimates compared to the measured masses. That is, an error of 100% indicates that the model mass is double that of the measured specimen. Similarly, negative percent errors indicate models with masses that are underestimates of the measured specimen, such that an error of −50% indicates a model that is half the mass of the measured specimen. Percent errors in mass are largely consistent within taxa, with extrema at each tail being comprised of Orthoptera (mean error, water‐filled: −57.21%; mean error, insect‐filled: −46.09%) and ladybirds (mean error, water‐filled: 202.03%; mean error, insect‐filled: 280.56%). Taxa with minimum percent errors are lacewings (mean error, water‐filled: −1.97%; mean error, insect‐filled: 23.52%), craneflies (mean error, water‐filled: 17.39%; mean error, insect‐filled: −47.91%) and honeybees/wasps (mean error, water‐filled: −8.70%; mean error, insect‐filled: −15.03%).

When taking specimen mass and aspect ratio (i.e., proportion of length to width) as fixed metrics for an equi‐mass prolate spheroid model, the resulting absolute size will often deviate from the measured specimens (Fig. 1C and D). As the aspect ratio is maintained as a constant, the percent errors shown in Figure 1C and D are identical for both body length and width. In this case, positive percent errors indicate equi‐mass models that result in size overestimates compared to the measured sizes. That is, an error of 100% indicates that the model size (i.e., length and width) is double that of the measured specimen. Similarly, negative percent errors indicate models with sizes that are underestimates of the measured specimen, such that an error of −50% indicates a model that is half the size of the measured specimen. Again, percent errors in size are largely consistent within taxa, with extrema at each tail being comprised of Orthoptera (mean error, water‐filled: 35.15%; mean error, insect‐filled: 25.13%) and ladybirds (mean error, water‐filled: −30.76%; mean error, insect‐filled: −35.90%) and minimum errors associated with lacewings (mean error, water‐filled: 0.89%; mean error, insect‐filled: −6.59%), craneflies (mean error, water‐filled: 3.30%; mean error, insect‐filled: −4.36%) and honeybees/wasps (mean error, water‐filled: 3.73%; mean error, insect‐filled: −3.96%).

For a prolate ellipsoid model, the body length, width and mass can all be fixed with respect to the specimen measurements and used to infer the specimen height (i.e. dorsoventral dimension). This additional degree of freedom should enable more realistic body geometry for emulating the specimen. This inferred body height can be compared to the body width that has served as a proxy for height in the previous models to see how the two compare (Fig. 1E and F). In this case, percent errors near zero indicate that ellipsoid heights are nearly equal to their widths, approximating spheroids. Non‐zero percent errors indicate ellipsoids that are increasingly different from their spheroidal approximations. In these cases, a positive percent error indicates an ellipsoid having larger inferred height than width, while a negative percent error indicates an ellipsoid having larger width than inferred height. Not surprisingly, this technique indicates that Orthoptera are the most vertically elongated of the taxa (mean error, water‐filled: 154.15%; mean error, insect‐filled: 101.71%), while ladybirds are the most horizontally elongated (i.e. flattened) taxa (mean error, water‐filled: −66.77%; mean error, insect‐filled: −73.62%). The most spheroidal taxa are lacewings (mean error, water‐filled: 3.04%; mean error, insect‐filled: −18.22%), pyralid/plutellid moths (mean error, water‐filled: −21.90.%; mean error, insect‐filled: −38.01%), craneflies (mean error, water‐filled: 22.47%; mean error, insect‐filled: −2.80%) and honeybees/wasps (mean error, water‐filled: 12.82%; mean error, insect‐filled: −10.46%).

### Comparison of radar cross‐sections

In addition to comparing the measured physical characteristics of the specimens against their modelled equi‐size, equi‐mass and ellipsoidal counterparts, we can also compare the measured and modelled radar cross‐sections. Table 1 shows summary error statistics for the along‐body and across‐body radar cross‐sections for each of the eleven taxon groups and all six model types. As with the statistics presented for the physical attributes, positive RCS percent errors represents a modelled RCS that is larger than the measured value and negative error values indicate that the modelled RCS is underestimated compared to the measurements (e.g., an error of 100% means that the modelled RCS is twice as large as the corresponding measurement, while an error of −50% indicates that the modelled RCS value is half of the measured value).

When comparing the results for models that differ only in internal composition, the insect‐paste‐filled models produce lower mean errors than the water‐filled models in almost all cases (Table 1). Moving from a composition of water to insect paste affects the modeled specimens in two ways: reducing the permittivity, which affects all three sets of models, and increasing the density, which affects only the equi‐mass and ellipsoidal models. The effect of the reduced permittivity can be seen by comparing the results of the two equi‐size models as these differ only in permittivity. Reducing the permittivity reduces the modelled RCS values, and since the modelled RCS is overestimated when compared to the measurements for most of the groups, reducing the permittivity has the overall effect of lowering the error in the RCS. For the equi‐mass models, moving from water to insect paste also has the effect of reducing the model sizes in all three dimensions. Similarly for the ellipsoidal models, increasing the density has the effect of reducing the size of the models in the dorsoventral axis. This reduction in model size compounds the effect of the reduced permittivity and further reduces the RCS compared to the equi‐size models leading to lower overall RCS errors values for these models. There are a few exceptions where the water‐filled models result in lower RCS errors than the insect‐paste‐filled models, but these reflect the limited cases in which the RCS is overestimated by the model compared to the measurements (e.g., the across‐body RCS for the pyralid/plutellid moth category, and the ladybird RCS using the equi‐mass model).

**Figure 2:**
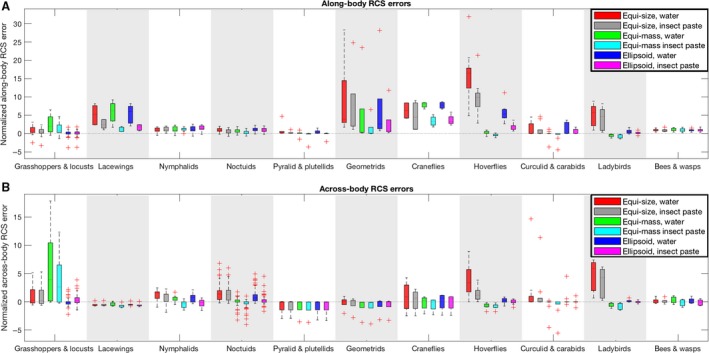
Normalized along‐body (A) and across‐body (B) RCS errors for each of the six models, separated by insect groups. The raw errors (modelled RCS value minus measured RCS value) were normalized by the corresponding mean measured along‐ or across‐body RCS for that insect group. RCS, radar cross‐section.

The RCS errors are also presented graphically in Figure 2, in which the error shown is the raw error (modelled value minus measured value) normalized by the mean RCS for each group to facilitate easier comparison among groups. Considering the water‐filled and insect‐paste‐filled models separately, the highest average RCS error for both of the internal materials is found to occur when the insects are represented using the equi‐size model (Table 1, Fig. 2). The mean along‐body RCS errors are lowest for the equi‐mass model, although this is likely biased by some of the groups (e.g., ladybirds and hoverflies) reporting negative along‐body RCS errors with the equi‐mass model. In terms of across‐body RCS the ellipsoidal model produces the lowest average errors for both the water and insect‐paste interior materials.

**Figure 3:**
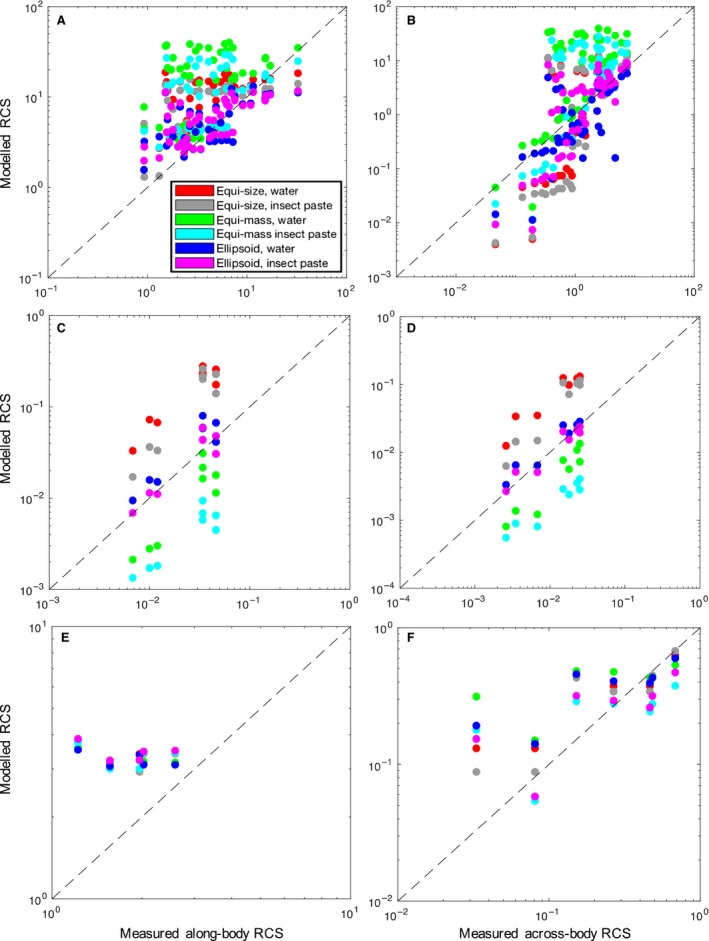
Comparisons between modelled and measured radar cross sections. Along‐body RCSs are shown in the left column and across‐body RCSs in the right column. The groups shown are Orthoptera (A, B), ladybirds (C, D) and Hymenoptera (E, F). RCS, radar cross‐section.

Three of the groups – Orthoptera, ladybird beetles and Hymenoptera – serve to illustrate the main cases of model performance. The modelled and measured RCSs for each specimen from these three groups are shown in Figure 3, with the along‐body RCSs shown in the left‐hand column and the across‐body RCSs in the right‐hand column. It is clear from Figure 3 that model performance varies across groups, with causes related to differences in the insects’ physical characteristics. For example, when modelling Orthoptera (Fig. 3, top row) the equi‐mass models (green and cyan) typically overestimate RCS, resulting in points above the one‐to‐one line. For ladybirds, however, the effect is reversed (Fig. 3, middle row), with equi‐mass models consistently underestimating RCS (green and cyan), resulting in points below the one‐to‐one line. In contrast, RCS values for the Hymenoptera (Fig. 3, bottom row) are very similar for all models, with all approximately falling near the one‐to‐one line. This ‘collapse’ of all six models to similar RCS values indicates that a prolate spheroid is a good approximation for the Hymenoptera, which validates the low physical errors seen for all models for this group in Figure 1. Conversely, wide variability of modeled RCSs for ladybirds and Orthoptera demonstrates that some model types do not accurately capture the scattering characteristics of these groups. Overall, the ellipsoidal models (Fig. 3, blue and magenta) are most accurate across all three groups. The only difference between the equi‐size and prolate ellipsoid models is the degree of freedom in varying the insect's height, yet we see significant differences in the resulting across‐body RCS values. This serves to demonstrate that knowledge of an insect's height is important even when considering a ventral look angle, as elongation in the dorsoventral axis will manifest in changes in both the along‐ and across‐body RCSs.

## Discussion

Routine radar monitoring of airborne animal migrations provides considerable benefits to a variety of stakeholders in society (Bauer et al. 2017). However, fully realizing these benefits necessitates improvements in our knowledge of radar‐scattering signatures of airborne animals. This is vital for enhancing interpretations of biological scatterers in the atmosphere in general, and for extracting information on the airborne insect fauna in particular. Here, we investigated the application of electromagnetic modelling using WIPL‐D software as a convenient way of creating radar cross‐sections for various flying insect taxa. The comparison of our results with laboratory measurements produced the following main findings.

In general, models using a prolate ellipsoid geometry were best able to emulate the measured radar cross‐sections across all taxa; there were clear additional improvements when using a homogenized insect‐paste instead of water to represent dielectric properties of the insect bodies. The performance of prolate spheroid models fell into three main categories. For taxa having naturally spheroidal body shapes, such as lacewings and Hymenoptera, the prolate models performed well. In these cases, the ellipsoid models assumed heights similar to their widths, thus approximating spheroids such that all six models produced similar RCS errors. Taxa with body shapes elongated in height and compressed in width, most notably locusts and grasshoppers (Orthoptera), produced consistent errors when using prolate spheroid models; the errors could, nonetheless, be accounted for by detailed consideration of typical body‐shapes of the taxa concerned. For example, when using an equi‐size model, height is underestimated by the orthopteran specimen width, resulting in underestimates of mass and RCS. When using an equi‐mass model with constant aspect, too much mass was distributed over the width and length, while not enough was distributed over the height, resulting in overestimates in overall size and RCS. Conversely, taxa with a flattened shape, such as ladybirds and hoverflies, had the opposite trends. When using an equi‐size model, the height is overestimated by the width, resulting in overestimates of mass and RCS. When using an equi‐mass model with constant aspect, too much mass is distributed over the height, while not enough is distributed over the width and length, resulting in underestimates in size and RCS. Most of the other taxa are less extreme examples of one of these three cases.

The results of our experiment show that it should be possible to input relatively simple physical measurements of insects, i.e., body length, width and height (which we have shown here to be important) into electromagnetic models; these will then able to produce reliable radar scattering cross‐sections for those taxa without the need for direct RCS measurements which require specialized equipment and are often experimentally difficult to perform. Improved accessibility of standardized RCS measurements from a large range of insect taxa will greatly advance the quantitative aspects of radar aeroecology, by allowing for more accurate estimation of aerial densities of the biological scatterers actually observed by weather surveillance radars.

In addition, the association of target properties with RCS size and shape values, achieved through calculations using electromagnetics theory, will be directly applicable to studies using special‐purpose entomological radars. For example, suppose an insect species is suspected to be migrating in a particular season (evidenced perhaps by its presence in trap catches), but the species is not among the few for which a measured RCS exists. Some simple measurements of its morphological dimensions combined with the modelling techniques described here would quickly provide an emulated target to compare with the ones detected by the ZLC entomological radar.

Further extension of this research could focus on incorporating the RCS values produced by electromagnetic modelling software with typical volume densities of airborne insects derived from aerial trapping studies. This would allow for the calculation of theoretical reflectivities for groups aerial fauna of known species composition and typical density, which could then be compared with reflectivities of biota recorded by weather surveillance radars. Applications such as these could serve to reveal the dominant taxa inhabiting the airspace from radar observations, while providing an avenue towards radar‐based classification methods, and represent a foundational step towards realizing the full potential of weather radar technology in entomology.

## Conflict of Interest

We declare that we have no competing interests.
